# Editorial: Updating amyloid neuropathy knowledge: from diagnosis to treatment

**DOI:** 10.3389/fneur.2023.1219317

**Published:** 2023-06-27

**Authors:** Isabel Conceição

**Affiliations:** ^1^Departamento de Neurociências e Saúde Mental, Centro Hospitalar Universitário Lisboa Norte (CHULN), Lisbon, Portugal; ^2^Faculdade de Medicina, Universidade de Lisboa, Lisbon, Portugal; ^3^Centro de Estudos Egas Moniz, Lisbon, Portugal

**Keywords:** ATTR amyloidosis, diagnosis, carpal tunnel syndrome, quality of life, neuropathic pain (NP)

Transthyretin amyloidosis (ATTR amyloidosis) is a rare, life-threatening, rapidly progressive, and heterogeneous disease caused by the accumulation of variant (ATTRv) or wild-type (ATTRwt) transthyretin amyloid fibrils in the heart, peripheral nerves, and other tissues and organs (1). While ATTRv amyloidosis has historically been considered primarily a neurological or cardiac disease, there has also been gradual description of a mixed phenotype and multisystemic involvement, depending on the TTR variant and various other factors (2).

Up to this point, over 140 TTR variants have been identified (3), with a wide range of phenotype/genotype correlations. According to the Transthyretin Amyloid Outcome Survey (THAOS) study, the Val30Met (p.Val50Met) genotype is the most predominant overall (4), with the exception of the United States, where the most prevalent TTR variant is Val122Ile (p.Val142Ile); more than 85% of individuals with this variant are African American (5). Shije et al. conducted a study to investigate the prevalence of the V122I mutation in African Americans with bilateral carpal tunnel syndrome (CTS) and to determine whether various demographic and clinical factors are associated with an increased likelihood of having the mutation. Despite the small sample size, the study found that 12.5% of the patients were V122I carriers, indicating that the frequency of the V122I mutation in this cohort may be higher than expected (3–4%). The presence of a family history of neuropathy, diagnosis of carpal tunnel syndrome after the 5th or 6th decade of life, and cardiomyopathy were factors associated with an increased likelihood of carrying the mutation. Periodic follow-up of these patients to rule out CTS, cardiomyopathy, or sensory disturbances in the extremities allows for earlier diagnosis and management of the disease, which improves outcomes in affected individuals.

In recent years, significant advances have been made in the diagnosis and treatment of ATTRv amyloidosis, allowing for the slowing of disease progression and preservation of quality of life (QOL) for those affected (6). Despite these advances, challenges remain in the diagnosis and management of ATTRv amyloidosis, particularly concerning long-standing disease with oculo-leptomeningeal manifestations that are not addressed by available disease-modifying therapies.

Although Magnetic Resonance Imaging (MRI) has been suggested to be the most reliable tool for identification of cerebral amyloid angiopathy (CAA) and meningeal amyloid deposits, its use is limited due to the high percentage of patients who have pacemakers. Cerebral amyloid angiopathy (CAA) is typically linked to specific MRI biomarkers indicating small-vessel brain damage, such as strictly lobar cerebral microbleeds, cortical superficial siderosis, centrum semiovale perivascular spaces, and white matter hyperintensities (7, 8). PET with an amyloid tracer ([11C] Pittsburgh compound B ([11C]PIB) or [18F] flutemetamol PET/CT) appears to be an attractive alternative for the detection of brain amyloid depositions in patients with ATTRv (9–11). A study by Uneus et al. explored the utility of a new imaging technique, [18F]flutemetamol PET/CT, for diagnosis and monitoring of CNS involvement in ATTR V30M amyloidosis patients. The major finding of the study was that amyloid deposition is mostly confined to the cerebellum. The study also found that amyloid deposition is related to age of disease onset but not to disease duration, in contrast with previously reported findings (9).

Periodic assessment of patients treated for polyneuropathy of ATTRv amyloidosis is mandatory in order to adjust therapy, delay clinical deterioration, and preserve their quality of life (QOL), as well as their social, economic, and psychological wellbeing (6).

A study by Di Stefano et al. showed that nerve conduction studies (NCSs) and handgrip strength (HGS) measurement could be useful tools for monitoring disease progression in patients with ATTRv amyloidosis, particularly those with major upper limb involvement such as carpal tunnel syndrome (12). The study found a positive correlation between compound muscle action potential (CMAP) of the median nerve and overall motor involvement, as measured by Neuropathy Impairment Score. Additionally, HGS measurement, which measures grip strength in the hand, was found to be a reliable indicator of overall hand motor strength and could be used to monitor disease progression over time. Assessing strength through additional HGS testing allows for increased sensitivity in comparison to the use of conventional NCSs, especially when motor nerves are not elicitable. The authors of this study suggested that a combination of NCSs and HGS measurements could constitute a simple and non-invasive way to monitor hand motor strength in patients with ATTRv amyloidosis and could be useful for assessment of disease progression and in guiding treatment decisions.

Symptom management is of major importance in ATTR amyloidosis due to its impact on patients' daily activities and QOL. One of the major symptoms is neuropathic pain caused by axonal peripheral neuropathy (12, 13). To treat neuropathic pain, drugs like serotonin-norepinephrine reuptake inhibitors (e.g., duloxetine or venlafaxine) or gabapentinoids (e.g., gabapentin or pregabalin) are the first-line therapies. As second-line options, weak opioid analgesics (e.g., tramadol or tapentadol) and topical agents (e.g., lidocaine or capsaicin) are recommended (6). Tozza et al. conducted a study to assess the prevalence of neuropathic pain in patients with late-onset ATTRv amyloidosis and in presymptomatic carriers. The study found that approximately 70% of late-onset ATTRv patients experienced neuropathic pain that worsened as peripheral neuropathy progressed and had a significant impact on patients' daily activities and quality of life. Those patients who experienced neuropathic pain were older; exhibited more severe disability, lower QoL, and significant autonomic involvement (according to the Compound Autonomic Dysfunction Test); and were more likely to have cardiomyopathy. Remarkably, the study also found that 8% of presymptomatic carriers reported neuropathic pain. This suggests that neuropathic pain may be an early symptom of ATTRv amyloidosis and may be worth monitoring in terms of disease progression and the identification of early manifestations of the disease.

In summary, all the articles and studies included in this Research Topic provide novel insights into diagnosis and disease monitoring, presenting ideas with the potential to improve management of ATTRv amyloidosis.

## Author contributions

The author confirms being the sole contributor of this work and has approved it for publication.
